# Toxic epidermal necrolysis induced by sintilimab in a patient with advanced non-small cell lung cancer and comorbid pulmonary tuberculosis: A case report

**DOI:** 10.3389/fimmu.2022.989966

**Published:** 2022-08-26

**Authors:** Gang Li, Sheng Gong, Ning Wang, Xiaojun Yao

**Affiliations:** ^1^ Department of Thoracic Surgery, The Public Health Clinical Center of Chengdu, Chengdu, China; ^2^ Department of Public Health, Chengdu Medical College, Chengdu, China

**Keywords:** immune-related adverse events (ir-AEs), neoadjuvant therapy, toxic epidermal necrolysis, non-small cell lung cancer, pulmonary tuberculosis

## Abstract

Immune checkpoint inhibitors (ICIs) have had a revolutionary effect on the treatment of patients with advanced non-small cell lung cancer (NSCLC), especially squamous cell lung cancer. However, ICIs may cause associated immune-related adverse events (ir-AEs). No case of sintilimab-induced toxic epidermal necrolysis (TEN) has been reported. In this report, we discussed a patient with advanced NSCLC and comorbid pulmonary tuberculosis who underwent immunotherapy and chemotherapy as neoadjuvant therapy and anti-tuberculosis therapy concurrently. Partial response (PR) of the tumor was achieved after three cycles of neoadjuvant therapy without cutaneous toxicities. Video-assisted thoracoscopic surgery (VATS) left lower lobectomy was performed successfully. Sintilimab and chemotherapy were administered as adjuvant therapy, after which the patient suffered severe TEN that rapidly progressed to cover >50% of the skin. TEN was associated with extensive rashes of the trunk and pruritus. With history of sintilimab use, clinical symptoms, and physical examination, TEN was diagnosed. Intravenous methylprednisolone and oral prednisone were administered until the patient totally recovered from the cutaneous toxicities caused by sintilimab. Monitoring of such rare but severe cutaneous toxicities is essential in patients who are treated with sintilimab.

## Introduction

With the wide spread use of immune check point inhibitors (ICIs) to treat the non-small cell lung cancer (NSCLC), immune-related adverse events (ir-AEs), known as toxicities associated with ICIs, during the treatment has been the focus of attention. Ir-AEs usually present as skin rash, thyroid dysfunction, hepatitis, colitis, and interstitial lung disease.

Cutaneous toxicities induced by ICIs account for approximately 33% all patients presenting with ir-AEs (1). Toxic epidermal necrolysis (TEN) is a cutaneous toxicity with detrimental effects on health. However, ICI-induced TEN has rarely been reported in literature. In this report, we describe a case of severe TEN that was induced by sintilimab after neoadjuvant therapy of a patient with advanced squamous cell-lung cancer and pulmonary tuberculosis.

## Case report

**Figure 1:**
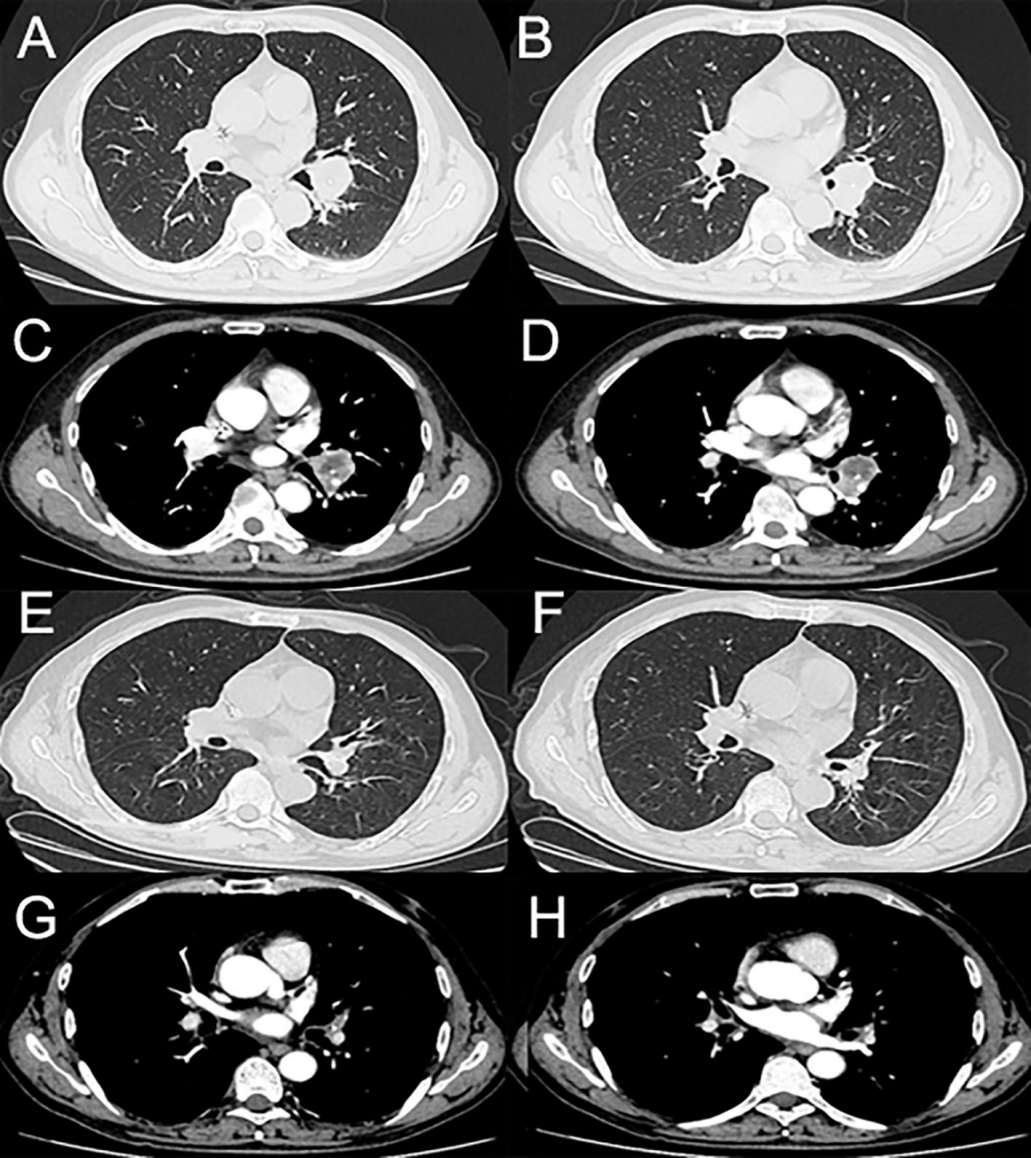
**(A–D)** The enhanced computed tomography (enhanced-CT) scans of the tumor before neoadjuvant therapy. **(E–H)** The enhanced-CT scans of the tumor after three cycles of neoadjuvant therapy.

A 59-year-old man was admitted to our hospital for the treatment of centrally located squamous cell-lung carcinoma and pulmonary tuberculosis. The computed tomography (CT) scans showed that the malignant lesion, located between the left upper and lower lobes, had invaded the left pulmonary artery (**Figure 1**). Pathological and etiological findings revealed a combined diagnosis of squamous cell-lung cancer (T3N0M0) and pulmonary tuberculosis. Anti-tuberculosis therapy, which including Isoniazid (300 mg per day), Rifampicin (450 mg per day), Ethambutol (750 mg per day), and Pyrazinamide (1250 mg per day), without any adverse events, was administered to cure the pulmonary tuberculosis. Neoadjuvant therapy, including sintilimab and conventional chemotherapy, was administered to achieve a complete response (CR) or partial response (PR). After three cycles of neoadjuvant therapy, the patient underwent video-assisted thoracoscopic surgery (VATS) left lower lobectomy, because the malignant lesion had shrunk and the left pulmonary artery had been isolated from the tumor. No cutaneous toxicity was observed during the first three cycles of neoadjuvant therapy. According to the post-operative pathologic results, the neoadjuvant therapy resulted in a PR. Therefore, the sintilimab and conventional therapy were continued as post-operative adjuvant therapy.

**Figure 2:**
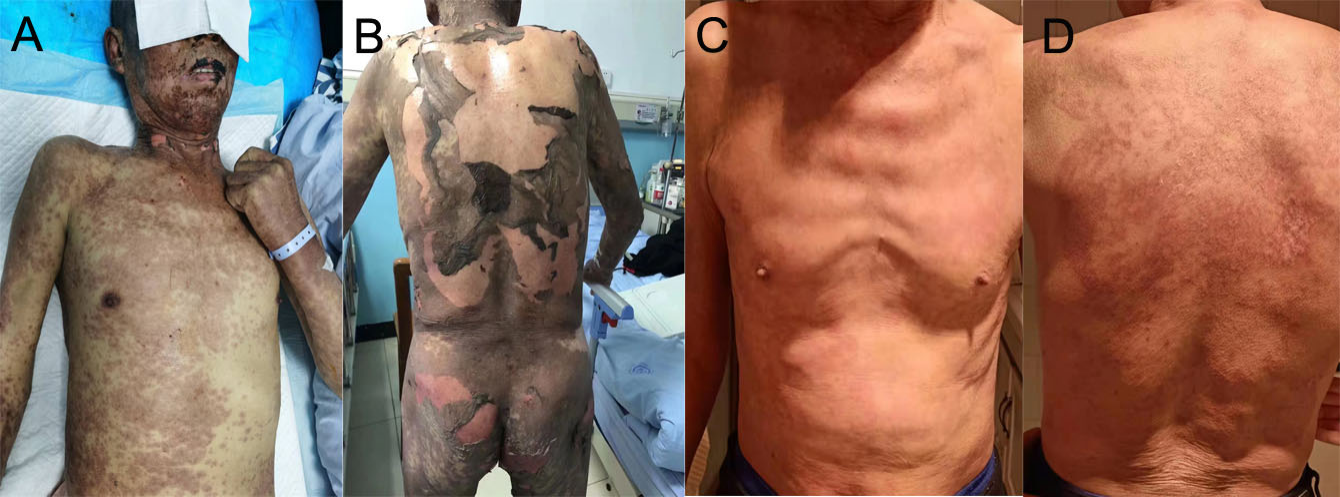
**(A, B)** TEN and rash induced by sintilimab in the patient with advanced NSCLC. **(C, D)** An image of the skin after recovery from severe TEN.

**Figure 3:**
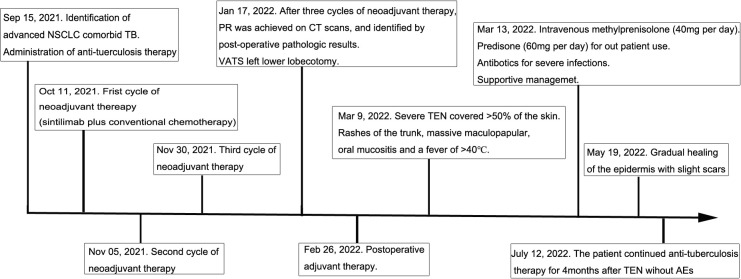
Anti-tuberculosis therapy: Isoniazid (300 mg per day), Rifampicin (450 mg per day), Ethambutol (750 mg per day), Pyrazinamide (1250 mg per day). Neoadjuvant therapy: Sintilimab (200 mg) plus platinum-based conventional chemotherapy (paclitaxel and cisplatin). PR, Partial response; TEN, Toxic epidermal necrolysis; AE, Adverse event.

Ten days after the post-operative adjuvant therapy, the patient suffered severe TEN, which rapidly progressed to cover >50% of the skin. TEN was associated with rashes of the trunk, pruritus, and a fever of >40 ℃. Massive maculopapular were observed in the chest and abdomen. Oral mucositis was also observed. The cutaneous lesions accounted for approximately 95% of the whole body surface (**Figure 2**). No pulmonary, gastrointestinal, or cardiac AEs were presented in this patient. Physical examinations showed the positive Nikolsky sign. Laboratory results showed that the neutrophil-to-lymphocyte ratio (NLR), platelet-to-lymphocyte ratio (PLR), C-reactive protein was 3.48, 217.39 and 53.28, respectively. The white blood cell was lower than normal because of chemotherapy. Due to financial constraints, no further skin biopsies were taken for pathological analysis. Because of the shortage of pathologic results, a dermatologist diagnosed TEN due to the history of sintilimab use, epidermal necrolysis, positive Nikolsky sign, high NLR and PLR, and so on. Exfoliative dermatitis, which was similar to TEN was suspected, but the Nikolsky sign was negative. Intravenous methylprednisolone (40 mg per day) was administered to this patient immediately. Additionally, prednisone (60 mg per day) was prescribed for out-patient use. Supportive management included wound care, nutritional supplementation, and analgesic use. Furthermore, Levofloxacin (500 mg per day) was administered to treat infections resulting from damage to the skin barrier. Four weeks after discharge from the hospital, we observed gradual healing of the epidermis with slight scars. Anti-tuberculosis therapy was continued for 4 months after diagnosis of TEN without any adverse events. The timeline of the treatment of the patient was presented in **Figure 3**.

## Discussion

Due to the widespread use of ICIs in the treatment of advanced NSCLC, ir-AEs become the focus of attention because the incidence and motility rates of ICI-induced severe ir-AEs were 4% and 0.34%, respectively (2). Therapeutic doses of ICIs and platinum-based chemotherapy could be used in the neoadjuvant therapy for NSCLC (3, 4). ICIs are relatively safe for patients with lung cancer and comorbid tuberculosis; however, associated ir-AEs should be monitored cautiously (5). In this case, under the concomitant administration of anti-tuberculosis therapy, the tumor shrunk in size after neoadjuvant therapy. Thereafter, the patient underwent lobectomy safely without any adverse events.

According to a study in a tertiary hospital, the incidence of sintilimab-induced ir-AEs was 29.03%, which was lower than that caused by nivolumab, pembrolizumab, or camrelizumab (6). The most common symptoms of ir-AEs were nausea and vomiting but not cutaneous toxicities (6). Cutaneous toxicities, including alopecia, pruritus and rash, are prevalent in ICI-induced ir-AEs during and/or after the treatment of anti-NSCLC (7, 8). Few studies have reported severe dermatitis events, such as Stevens-Johnson Syndrome/Toxic epidermal necrolysis (SJS/TEN) (9–11) and severe oral mucositis (12). However, no case of severe sintilimab-induced TEN has been reported. In this case, severe TEN was almost fatal. The algorithms for causality defined the sintilimab in the categories “Probable” for this patient using the ALDEN causality scores (11, 13). The score of sintilimab was higher than that of the other medicines in the ALDEN causality score criterion. So we considered that the TEN was induced by sintilimab in this patient. Using the Common Terminology Criteria (CTC) for Adverse Events 5.0, grade 4 ir-AEs cutaneous toxicity was diagnosed.

Several predictive risk factors were associated with ICI-induced ir-AEs in the treatment of advanced NSCLC. According to previous reports, first-line treatment with ICIs and lung immune prognostic index (LIPI) were independent predictive risk factors for ir-AEs in patients with advanced NSCLC (14). Furthermore, concomitant chemotherapy use, high body mass index (BMI), and presence of epidermal growth factor receptor (EGFR) mutation were significant predictors for ir-AEs (15). BP180-specific IgG was associated with skin-adverse events in a histological study (16). Additionally, ICI combined with radiotherapy may cause severe skin-associated adverse events (17). For medicines that caused SJS/TEN more often than ICIs, no NSAIDs, anti-epileptic drugs, sulfa drugs, and allopurinol were used before and during the whole treatment. In this case, sintilimab was used as first-line treatment medication. However, the patient had a low BMI of 17.6 kg/m^2^, which was not matched the previous risk factors. So debate was existed about the causality of the drug-induced clinical events. Hypersensitivity reactions of drugs may claim the reason of such reaction clinical symptoms (18). EGFR gene mutations and anaplastic lymphoma receptor factor (EGFR) were not evaluated because of the lower likelihood of those mutations in squamous cell carcinomas. Additionally, programmed cell death-1/programmed cell death-ligand 1 (PD-1/PD-L1) was not assessed due to patient’s financial constraints. Since, TEN was related to ICI use as first-line treatment. Monitoring of the presence of severe cutaneous caused by ICIs was essential.

Appropriate treatment for TEN is crucial for patients with severe cutaneous toxicities, because ICI-induced TEN may be fatal during or after the treatment of advanced NSCLC. Methylprednisolone is the key regimen in the treatment of cutaneous toxicities, including rashes, bullous lesions, hypertrophic nodules, eruptive keratoacanthoma, papules, and plaques (19, 20). The patient recovered approximately 1 month post-discharge from the hospital after methylprednisolone was administered.

## Limitations

Because skin biopsies were not conducted to this patient, diagnosis of TEN was mainly based on administration of sintilimab, physical examination, and laboratory results. It is the limitation of this manuscript.

## Conclusion

Various forms of ICI-induced cutaneous toxicities during the treatment of advanced NSCLC have been reported. This case report was the first to describe severe TEN with associated extensive rashes of the trunk, limbs, and buccal mucosa 6 months after the initiation of sintilimab. Monitoring such rare and severe cutaneous toxicities is essential in patients treated with sintilimab. Further research is warranted for the incidence, pathophysiology, and mechanism of the ir-AEs associated with ICIs.

## Data availability statement

The raw data supporting the conclusions of this article will be made available by the authors, without undue reservation.

## Ethics statement

The studies involving human participants were reviewed and approved by the Ethical Review Committee for Research in The Public Health Clinical Center of Chengdu. The patients/participants provided their written informed consent to participate in this study. Written informed consent was obtained from the individual(s) for the publication of any potentially identifiable images or data included in this article.

## Author contributions

GL, SG, and NW drafted the manuscript and obtained the image. XY supervised the manuscript writing. All the authors reviewed the manuscript and approved the final version.

## Funding

The author(s) received no financial support for the research, authorship, and/or publication of this article.

## Acknowledgments

The authors are grateful for the assistance provided by LiangShuang Jiang and other staff at Chengdu Public Health Clinical Center in preparing this manuscript.

## Conflict of interest

The authors declare that the research was conducted in the absence of any commercial or financial relationships that could be construed as a potential conflict of interest.

## Publisher’s note

All claims expressed in this article are solely those of the authors and do not necessarily represent those of their affiliated organizations, or those of the publisher, the editors and the reviewers. Any product that may be evaluated in this article, or claim that may be made by its manufacturer, is not guaranteed or endorsed by the publisher.
